# Age-dependent prevalence of 14 high-risk HPV types in the Netherlands: implications for prophylactic vaccination and screening

**DOI:** 10.1038/sj.bjc.6604162

**Published:** 2008-01-08

**Authors:** V M H Coupé, J Berkhof, N W J Bulkmans, P J F Snijders, C J L M Meijer

**Affiliations:** 1 Department of Clinical Epidemiology and Biostatistics, VU University Medical Centre, PO Box 7057, Amsterdam 1007 MB, The Netherlands; 2 Department of Pathology, VU University Medical Centre, PO Box 7057, Amsterdam 1007 MB, The Netherlands

**Keywords:** human papillomavirus, age-dependent prevalence, vaccination, screening

## Abstract

We determined the prevalence of type-specific hrHPV infections in the Netherlands on cervical scrapes of 45 362 women aged 18–65 years. The overall hrHPV prevalence peaked at the age of 22 with peak prevalence of 24%. Each of the 14 hrHPV types decreased significantly with age (*P*-values between 0.0009 and 0.03). The proportion of HPV16 in hrHPV-positive infections also decreased with age (OR=0.76 (10-year scale), 95% CI=0.67–0.85), and a similar trend was observed for HPV16 when selecting hrHPV-positive women with cervical intraepithelial neoplasia grade 2 or worse (CIN2+) (OR=0.76, 95% CI=0.56–1.01). In women eligible for routine screening (age 29–61 years) with confirmed CIN2+, 65% was infected with HPV16 and/or HPV18. When HPV16/18-positive infections in women eligible for routine screening were discarded, the positive predictive value of cytology for the detection of CIN2+ decreased from 27 to 15%, the positive predictive value of hrHPV testing decreased from 26 to 15%, and the predictive value of a double-positive test (positive HPV test and a positive cytology) decreased from 54 to 41%. In women vaccinated against HPV16/18, screening remains important to detect cervical lesions caused by non-HPV16/18 types. To maintain a high-positive predictive value, screening algorithms must be carefully re-evaluated with regard to the screening modalities and length of the screening interval.

Infection with high-risk human papillomavirus (hrHPV) is the necessary cause of cervical cancer (Munoz *et al*, 2003). Prophylactic vaccines are now available that are effective against incident and persistent HPV16 and 18 infections (Koutsky *et al*, 2002; Munoz *et al*, 2003; Harper *et al*, 2006; Villa *et al*, 2006). Mass vaccination can have a substantial impact on the cervical cancer incidence, even in developed countries where the incidence is already low because of implementation of organised cervical screening. To make a well-judged decision about the future role of both vaccination and screening in cervical cancer prevention, detailed information about type-specific hrHPV distribution is required. Data on hrHPV prevalence and type distribution in the Netherlands have been reported previously, but only for a relatively small cohort of 3305 women (Jacobs *et al*, 2000a; Clifford *et al*, 2005). To obtain reliable estimates of the hrHPV type distribution in relation to age, both for women with normal and for women with abnormal cytology, data from a far larger cohort are needed. In this study, we determined the age-dependent prevalence of 14 hrHPV types and the age-dependent type distribution within hrHPV-positive women from a cohort of 45 362 women in the Netherlands aged 18–65 years. The results from the current study give an impression of the potential benefits that can be achieved from HPV16/18 vaccination. They can also be used to gain insight into the effects of partial cross-protection against HPV types 31 and 45 (Harper *et al*, 2006). Furthermore, the figures will serve as inputs for simulation models in which different vaccination and screening strategies will be compared.

## MATERIALS AND METHODS

### Data

Data from 45 362 Dutch women between 18 and 65 years of age were collected. Cohort participants were recruited from GP practices in the larger Amsterdam area between 1999 and 2002. Women between 29 and 61 years of age (*n*=44 102) participated in the POBASCAM study, a randomised controlled population-based screening trial evaluating the implementation of hrHPV testing in cervical screening (Bulkmans *et al*, 2004). The POBASCAM trial was approved by both the Medical Ethics Committee of the VU University Medical Center (no. 96/103) and the Ministry of Public Health (VWS no. 328650) and has been registered as an International Standard Randomised Controlled Trial (ISRCTN20781131). Women younger than 29 years (*n*=1109) or older than 61 years (*n*=151) obtained a cervical smear for opportunistic screening. All women underwent cytological testing as well as hrHPV testing. Women with abnormal cytology or cervical intraepithelial neoplasia (CIN) in the previous 2 years were excluded.

### Cytology, hrHPV DNA testing, genotyping

Conventional cytological smears were collected and read according to the CISOE-A classification used in the Netherlands. Translation into the Bethesda 2001 classification is easy (Bulk *et al*, 2004). Cytological results were grouped as normal or borderline mild dyskaryosis (BMD), and >BMD. Cervical scrapes were tested for the presence of hrHPV DNA using the consensus GP5+/6+-PCR-EIA method, which detects 14 hrHPV types (HPV16, 18, 31, 33, 35, 39, 45, 51, 52, 56, 58, 59, 66, and 68) (Jacobs *et al*, 1997). hrHPV testing was blinded to the cytological result. hrHPV-positive samples were subjected to hrHPV typing by reverse line blot analysis of PCR products (van den Brule *et al*, 2002). Of screening-eligible women who participated in the POBASCAM trial (aged 29–61 years), histological results of biopsy specimen taken at colposcopy inspection were included. All cases of CIN were included that were found after the referral smear (baseline smear or repeat smear at 6 or 18 months) and within 3 years after baseline. Cases were classified as CIN 1, 2, or 3 or invasive cancer in the local hospital and were not subjected to revision.

### Statistical methods

Age-specific estimates of the hrHPV prevalence (10-year age groups) were computed for all women and for women with normal cytology. Overall and type-specific hrHPV prevalences were related to age by logistic regression analyses. To obtain smooth functions of age, cubic splines with knots at age 30 and 50 years were used (Wold, 1974). The uncertainty about the estimated splines was represented by 99% point wise correct confidence intervals. Within the subset of hrHPV-positive women, the effect of age on the type distribution was assessed by logistic regression analyses. In statistical tests, only screening-eligible women (age 29–61 years) were included. Separate analyses were performed for women without confirmed CIN2+ and for women with confirmed CIN2+. The analyses were repeated for women with single infections.

With vaccination in mind, particular attention was paid to HPV types 16, 18, 31, and 45. Types 31 and 45 are of interest because vaccination against 16/18 may result in partial immunity for 31 and 45 through cross-protection (Harper *et al*, 2006). Within the group of screening-eligible hrHPV-positive women, we estimated the type distribution of HPV16, 18, 31, 45 *vs* other types. Separate calculations were carried out for women without confirmed CIN2+ and women with confirmed CIN2+. This was carried out by hierarchically assigning women to (1) HPV16, (2) HPV18 without the presence of HPV16, (3) HPV31 or 45 without HPV16 or 18, or (4) none of the HPV types 16, 18, 31, or 45. This means that, for example, assignment to HPV16 was carried out independently of whether a woman was simultaneously infected with other HPV types. Analyses were repeated for women with single infections.

Vaccination against HPV types 16 and 18 will affect current screening practice, because a decrease in the prevalence of high-grade CIN is expected to lead to a decrease in the positive predictive value of a screening instrument. To gain insight into the potential effects of vaccination on the predictive value of a positive screening test, we computed the positive predictive values for detection of CIN2+ for women without HPV16 and 18. We computed separate predictive values of positive cytology, a positive HPV DNA test, and a double-positive test (positive HPV test and a positive cytology). Notably, the double-positive test result is a prerequisite for colposcopy referral in a screening setting where cytology is used as a triage tool in HPV-positive women. The analyses were repeated for women without HPV16, 18, 31, and 45. The positive predictive values were adjusted for women who did not attend repeat screening by Kaplan–Meier estimation.

## RESULTS

Table 1 shows the age-specific estimates of the hrHPV prevalences, for all women combined and for women with normal cytology. Table 1 shows the number of women in each age group who are infected with each of the 14 HPV types. The overall hrHPV prevalence was 5.6%. In women with normal cytology, the hrHPV prevalence was 4.0%. HPV16 was the most common type (1.8% in all women, 1.1% in normal cytology), followed by types 31 and 18. The hrHPV prevalence in all women aged 29–61 years decreased significantly with age for all hrHPV types (*P*-values between 0.0009 and 0.03). Significance could also be demonstrated when selecting only those women with normal cytology.

**Figure 1:**
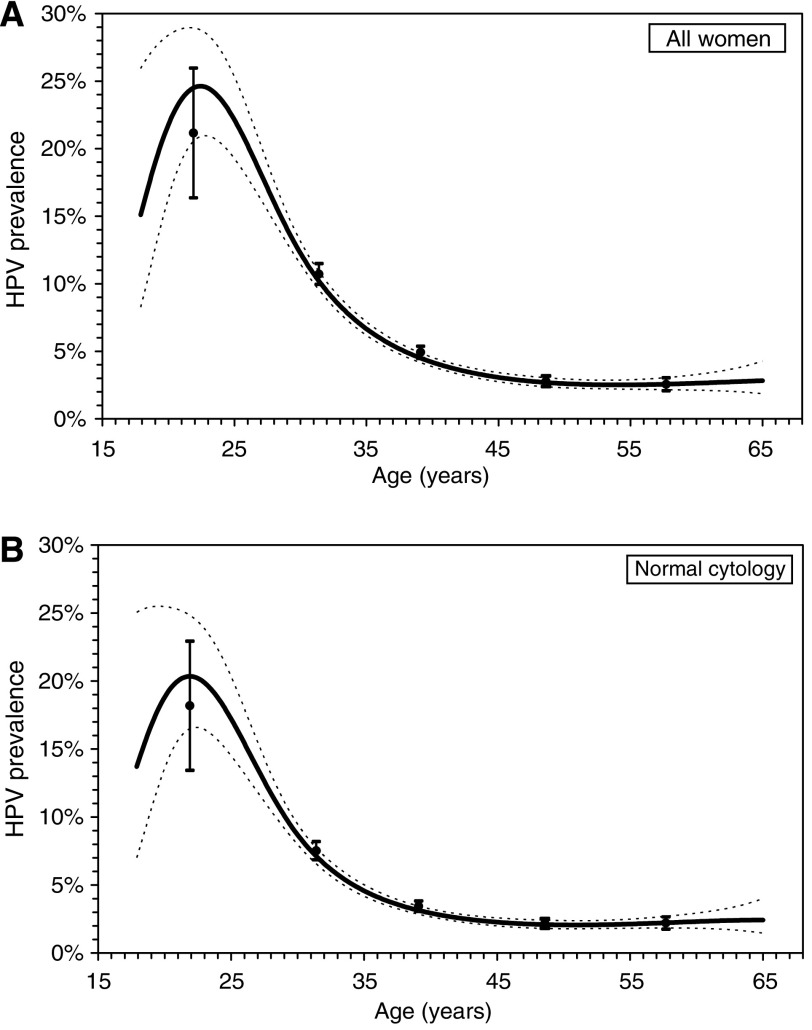
The relationship between hrHPV prevalence and age for all women (*n*=45 362) (**A**) and for women with normal cytology (*n*=43 737) (**B**).

**Figure 2:**
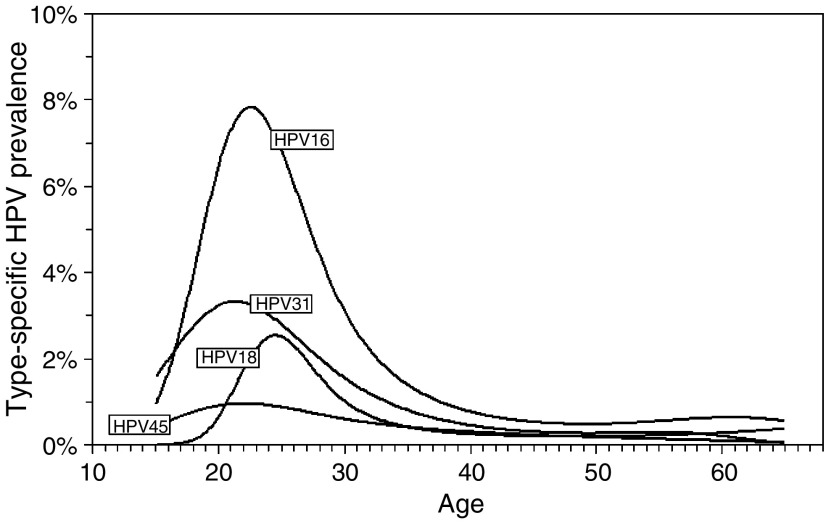
The relationship between the prevalence of HPV types 16, 18, 31, and 45 and age for women with normal cytology (*n*=43 875).

Figure 1 shows the age-dependent overall hrHPV prevalence for all women irrespective of cytological status (Figure 1A) and for women with normal cytology (Figure 1B). The dashed lines represent the lower and upper 99% confidence bands of the fitted curves. Age-specific estimates (10-year age groups) and 99% confidence intervals were also included in the figures. Overall hrHPV prevalence peaked at 22 years, with a peak prevalence of 24%. Beyond 45 years, the hrHPV prevalence reached a plateau of 3%. For normal cytology, a similar pattern was observed with a slightly lower peak prevalence of 20% (Figure 1B). The fitted curves lie within the 99% confidence intervals of the age-specific estimates. Because only a small sample of young women (18–24 years) was included in the data, wide confidence intervals were found in this age group. Figure 2 shows separately fitted prevalence curves for hrHPV types 16, 18, 31, and 45 in women with normal cytology. Again, the functions were single-peaked with peaks before age 30 years.

**Figure 3:**
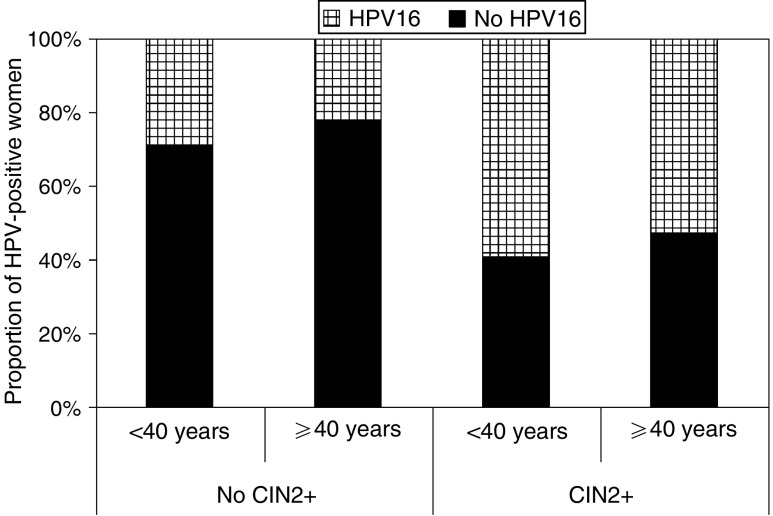
The proportion of HPV16 in hrHPV infections in women younger than 40 years of age and older women. Results are given for women without confirmed CIN2+ and for women with confirmed CIN2+.

Within the set of hrHPV-positive screening-eligible women (age 29–61 years) with a valid HPV typing result, the proportion of HPV16 infections decreased significantly with age (OR=0.76 (10-year scale), 95% CI=0.67–0.85, *P*<0.001). For HPV39 and HPV52, a marginal decrease with age was found as well (both *P*-values 0.03). In the subset of women with confirmed CIN2+, the association between HPV16 and age was in the same direction (OR=0.76, 95% CI=0.56–1.01, *P*=0.06). No relationship between hrHPV type and age was observed for any of the other types. The impact of age on the proportion of HPV16 infections in hrHPV-positive women is illustrated in Figure 3. In women with confirmed CIN2+, the proportion of HPV16 infections decreased from 59% in women younger than 40 years to 53% in older women. The results for women with single infections were nearly identical.

**Figure 4:**
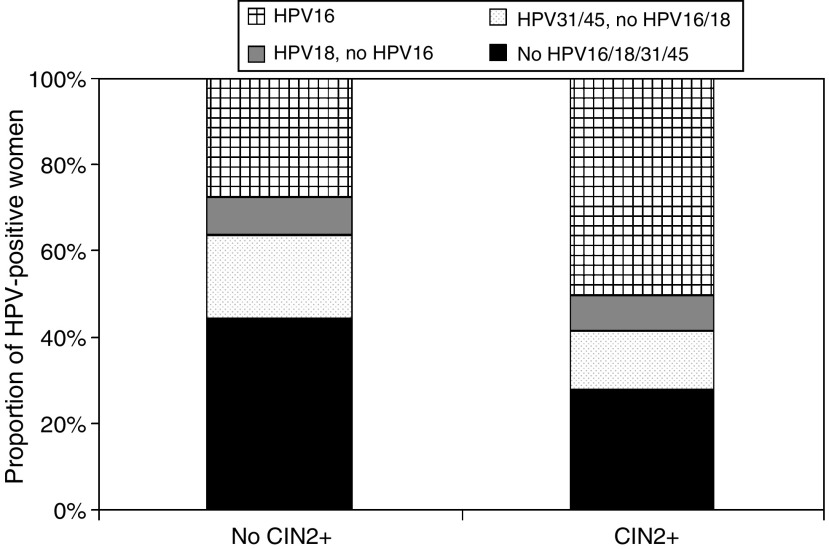
The HPV type distribution in hrHPV-positive women eligible for routine screening. The type distribution is shown separately for women without confirmed CIN2+ and women with confirmed CIN2+. hrHPV-positive women were classified as infected with (1) HPV16, (2) HPV18 without presence of HPV16, (3) HPV31 or 45 without HPV16 nor HPV18, or (4) none of the HPV types 16, 18, 31, or 45.

In hrHPV-positive women who were eligible for routine screening, 33% was infected with HPV16, 9% was infected with HPV18 without 16, and 59% was infected with HPV16, 18, 31, and/or 45. HrHPV infections with types other than HPV16, 18, 31, and/or 45 were found in 41% of the women. Figure 4 shows the type distribution in screening-eligible hrHPV-positive women with confirmed CIN2+ and in women without confirmed CIN2+. In women with confirmed CIN2+, HPV16 and/or HPV18 was found in 65% of the women. Women with infections other than HPV16, 18, 31, or 45 comprised 24% of the women with confirmed CIN2+. The type distribution in women with single infections was very similar.

**Figure 5:**
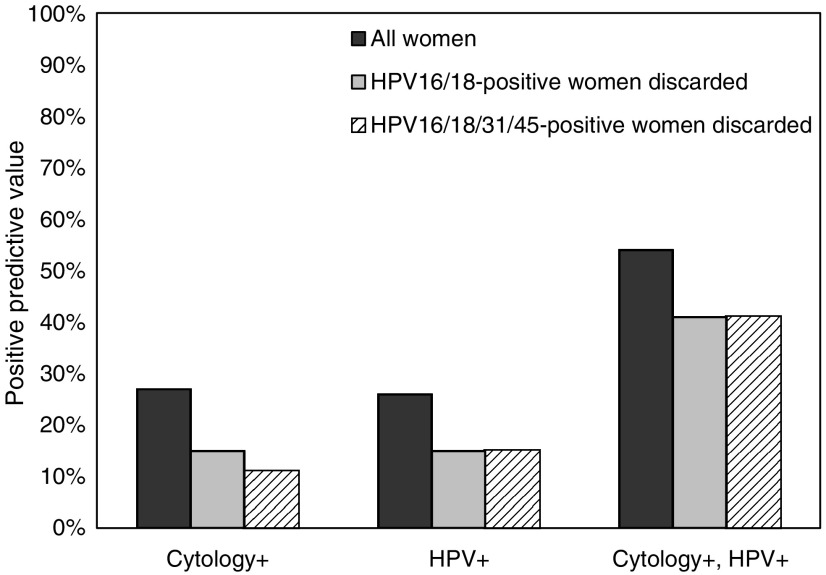
The positive predictive values for detection of CIN2+ lesions in women aged 29–61 years eligible for routine screening in case screening had been carried out by means of cytology, HPV DNA testing, or both. Results are shown for the current data set (black bars), when excluding HPV16/18-positive infections from the data (grey bars), and when excluding HPV16/18/31/45-positive infections (striped bars).

Figure 5 shows the positive predictive values for detection of CIN2+ lesions in women aged 29–61 years eligible for routine screening, in case screening is carried out by means of cytology, HPV testing or both cytology and HPV testing. The black bars represent the predictive values for the current HPV type distribution. The predictive values of abnormal cytology and a positive hrHPV test for detection of CIN2+ were 27 and 26%, respectively. The predictive value of positive results on both tests was 54%. When excluding HPV16- and HPV18-positive infections from the data, the predictive values of positive cytology, positive hrHPV test, and double-positive tests decreased to 15, 15, and 41%, respectively. When excluding all HPV16/18/31/45-positive infections, the positive predictive values became 11, 15, and 41%, respectively.

## DISCUSSION

In this study, data from a cohort of 45 362 Dutch women between 18 and 65 years of age were used to estimate the relationship between type-specific hrHPV prevalence and age in the Netherlands. We have shown that the prevalence of hrHPV peaks between 20 and 25 years, and we estimated the peak prevalence at 24%. Furthermore, we have shown that the proportion of HPV16 infections in hrHPV-positive smears decreases with age. We estimated that around 65% of hrHPV-positive women with a confirmed CIN2+ have an HPV16 and/or 18 infection. This indicates that non-HPV16/18 infections remain important with HPV16/18 vaccination.

The data set consisted of women aged 29–61 years eligible for routine screening and women younger than 29 or older than 61 years outside the routine screening age range. The hrHPV prevalence in women outside the routine screening age range may be biased because they visited their GP on own initiative. However, comparison of the HPV prevalence in opportunistically screened women aged 27 or 28 years to screening-eligible women aged 29 or 30 years did not show a trend shift in HPV prevalence (data not shown). Moreover, we observed an overall hrHPV prevalence of 21% in the age group 18–24 years, which is consistent with the international literature (Winer *et al*, 2003; Manhart *et al*, 2006; Cuzick *et al*, 2006).

Regarding the relationship between the hrHPV prevalence and age, we observed high prevalences at young age, and a subsequent decrease until 45 years of age. Above 45 years, the hrHPV prevalence reached a plateau level. The decrease in hrHPV prevalence was also observed for each hrHPV type separately. This decrease may be related to several factors that have been discussed in detail in the literature (Jacobs *et al*, 2000b; Castle *et al*, 2006). The observed relationship between hrHPV prevalence and age is in agreement with data from several European and North American studies (Jacobs *et al*, 2000a; Sellors *et al*, 2000; de Sanjose *et al*, 2003; Castle *et al*, 2005; Ronco *et al*, 2005; Manhart *et al*, 2006). In our large data set, we did not find a second hrHPV peak at age beyond 45 years as detected in several Latin American countries (Franceschi *et al*, 2006). Within hrHPV-positive women, HPV16 and HPV31 were the most prevalent types. Both types were also the most prevalent ones in an earlier pooled analysis of data from three European countries (Clifford *et al*, 2005).

A remarkable finding of this study is the negative association between the prevalence of HPV16 in hrHPV infections and age. A similar negative association was found in women eligible for routine screening with a confirmed CIN2+ lesion. For the other HPV types in hrHPV infections, associations with age were zero or minor. Although, in absolute sense, the decrease in HPV16 infections with age was small, we think that our observation reveals an interesting effect of cervical screening. HPV16 infections have a higher chance of progressing to cervical lesions than other infections; therefore, HPV16 infections also have a higher chance of being detected during the first round of routine cytological screening. In that case, the proportion of HPV16 infections will decrease at older age because the lesions containing the HPV16 infection have been eradicated.

From our results, it can be inferred that the maximum reductions in hrHPV infections and in CIN2+ lesions that can be achieved by prophylactic HPV16/18 vaccination would be about 42 and 65%, respectively. When an HPV16/18 vaccine would also offer full cross-protection against types 31 and 45 (Harper *et al*, 2006), the maximum reductions in hrHPV infections and in CIN2+ lesions would be about 60 and 76%, respectively. In reality, the reductions will be smaller because women with an HPV16/18/31/45-positive infection may be co-infected with another type. To summarise, screening remains necessary to detect at least 20–30% of the CIN2+ cases that cannot be prevented by prophylactic vaccination.

Implementation of mass vaccination may affect screening programmes. The primary aim of screening in a vaccinated population is to detect (pre-)cancerous lesions caused by non-HPV16/18 infections. We showed that the positive predictive values of cytology and HPV testing decrease when HPV16/18 infections have been removed from the data. The main reason for this is that HPV16 has a relatively high prevalence in hrHPV-positive women with confirmed high-grade CIN. This diminishes the effect of cervical screening on the incidence of cervical cancer and may lead to a discussion about the cost-effectiveness of cervical screening in vaccinated women. Although formal modelling analyses are required to evaluate the cost-effectiveness of screening strategies with vaccination, changes in the length of the screening interval and/or the age at which screening starts can be anticipated. A related discussion concerns the choice of the primary screening instrument. Meta-analyses have shown that, HPV DNA testing has a higher sensitivity than cytology (Arbyn *et al*, 2006; Cuzick *et al*, 2006) and may be implemented in cervical screening in the future. In this study, we have shown that on removing HPV16/18 infections, the positive predictive values of HPV testing and cytology show similar decreases. However, in a population vaccinated against HPV16/18, additional factors may cause a further degradation of the positive predictive value of cytology (Franco *et al*, 2006). More specifically, a decreasing prevalence of high-grade CIN may make it increasingly more difficult to differentiate abnormalities in cervical smears. On the other hand, we also computed that a high positive predictive value can still be achieved when cytology is applied only as a triage tool in HPV-positive women. To illustrate, we computed a CIN2+ detection rate of about 40% in hrHPV-positive women with abnormal cytology after removing all HPV16/18/31/45 infections. Such preliminary calculations indicate that screening in women vaccinated for HPV16/18 can still be performed with maintenance of the CIN2+ detection rate after referral. However, screening algorithms must be carefully re-evaluated with regard to the choice of the primary and secondary screening modality and length of the screening interval.

## Figures and Tables

**Table 1:** The hrHPV prevalences in 10-year age-groups, for all women combined (*n*=45 362) and for women with normal cytology (*n*=43 737)

**Age-group**	***n***	**Total hrHPV (%)**	**HPV16**	**HPV18**	**HPV31**	**HPV33**	**HPV35**	**HPV39**	**HPV45**	**HPV51**	**HPV52**	**HPV56**	**HPV58**	**HPV59**	**HPV66**	**HPV68**
*All women*																
18–24	482	102 (21.2)	36	11	15	6	4	6	3	13	8	14	4	5	14	0
25–34	10 828	1161 (10.7)	419	127	174	76	48	67	78	93	93	79	65	26	76	17
35–44	15 303	753 (4.9)	235	60	112	48	39	39	65	46	36	52	53	14	30	15
45–54	11 556	321 (2.8)	80	37	36	18	12	8	22	21	19	34	16	8	24	1
55–65	7193	184 (2.6)	49	14	23	14	10	6	6	15	7	20	11	5	10	4
																
Total	45 362	2521 (5.6)	819	249	360	162	113	126	174	188	163	199	149	58	154	37
																
*Women with normal cytology*																
18–24	440	80 (18.2)	31	6	11	4	4	5	3	9	5	11	2	4	9	0
25–34	10 238	770 (7.5)	246	81	127	38	31	44	50	62	55	53	41	18	60	9
35–44	14 751	508 (3.4)	132	39	76	31	29	25	48	26	23	39	35	13	19	11
45–54	11 239	244 (2.2)	52	26	26	13	7	5	19	10	17	30	11	8	19	1
55–65	7069	156 (2.2)	41	11	19	11	10	6	6	9	5	20	10	5	10	4
																
Total	43 737	1758 (4.0)	502	163	259	97	81	85	126	116	105	153	99	48	117	25

In each age group, the number of women that is infected with each of the 14 HPV types is shown.
